# Workplace violence and burnout among emergency medical service workers and nurses in Germany: a cross-sectional study

**DOI:** 10.1186/s12960-025-01026-y

**Published:** 2025-11-20

**Authors:** Karsten Roth, Julia Köppen, Cornelia Henschke

**Affiliations:** 1 Department of Health Care Management, Berlin Centre of Health Economics Research (BerlinHECOR), Berlin University of Technology, Straße des 17. Juni 135, H80, 10623 Berlin, Germany; 2 Institute of General Practice and Interprofessional Care, University Hospital Tübingen, Tübingen, Germany; 3 Department of Health Services Research and Nursing Science, Bielefeld University, Bielefeld, Germany

**Keywords:** Burnout, Violence, Abuse, Nurses, Paramedics, Satisfaction

## Abstract

**Background:**

Nonphysician healthcare workers play a crucial role in patient care, often under challenging conditions. Workplace violence puts professionals such as nurses and emergency medical service (EMS) workers at risk for (emotional) stress. This study comparatively analyzed the burden of workplace violence, burnout risk, and their associations among nurses and EMS workers.

**Methods:**

Two surveys were conducted using (i) a paper–pencil questionnaire for nurses and (ii) an online questionnaire for EMS workers in 2015. The surveys assessed experiences of workplace complaints, violence, and burnout risk measured by the Maslach Burnout Inventory (MBI). Data were analyzed descriptively (frequencies, means, percentages, Pearson correlation coefficients). Associations between workplace violence and burnout were estimated using binary logistic regression, adjusting for gender, employment status, work experience, education, and working conditions.

**Results:**

Data from 3,169 nurses (84.7% female) and 1,024 EMS workers (13.6% female) were analyzed. Frequent verbal abuse—ranging from daily incidents to several times a month—was reported by 44.7% of nurses and 59.9% of EMS workers, while 12.2% and 23.5%, respectively, experienced physical violence from patients or their families. Overall, a large proportion of employees in both professions have a moderate to high risk of burnout in the dimension of emotional exhaustion (EE) (nurses: 58.1%, EMS workers: 56.3%) and in the dimension of depersonalization (DP) (nurses: 58.4%, EMS workers: 74.4%). Logistic regression revealed that physical violence was significantly associated with a high risk of burnout in the dimensions EE and DP in nurses, and EE in EMS workers. Verbal abuse by patients was significantly associated with EE and DP in nurses, and with DP in EMS workers. In addition, an increased risk of burnout in both MBI dimensions was significantly associated with variables of working conditions and work experience for both professions.

**Conclusions:**

This is the first comparative study to examine the association between workplace violence and the risk of burnout among nurses and EMS workers. These findings highlight the need for strengthened measures to address workplace violence, prevent burnout among employees, and support staff in coping with these challenges. Improvements in working conditions must also be considered.

**Supplementary Information:**

The online version contains supplementary material available at 10.1186/s12960-025-01026-y.

## Background

Workplace violence against health care professionals perpetrated by patients and visitors has been a persistent and growing problem worldwide, with the health sector being at major risk due to the fundamental nature of the services delivered and the current work environment [1]. Although there is no agreed definition of workplace violence across countries, violent behavior in which staff are abused, threatened, or assaulted in circumstances related to their work, can be categorized into physical and psychological violence, with the latter receiving increasing attention in recent years [2, 3]. Experiencing such violence creates stress and can lead to frustration and anger, but also to more severe consequences such as burnout. This, in turn, can affect the delivery of health services, e.g., by reducing the quality of services provided or by health workers deciding to leave the health profession [3].

Large-scale reviews have shown that workplace violence is a widespread phenomenon across various healthcare professions and regions. For example, a systematic review of 65 studies involving 61,800 health care workers across 30 countries found that approximately 19% had experienced physical workplace violence within the past year [1]. In a larger review covering 253 studies with 331,544 healthcare workers, 61.9% reported experiencing some form of workplace violence. Among these, 42.5% experienced non-physical violence and 24.4% physical violence [4]. The data indicate variation by occupational group: nurses reported the highest exposure rate at 59.2%, followed by physicians (56.8%) and other health professionals (44.4%). This suggests that nurses are 1.3 times more likely to experience workplace violence than other health professionals.

Other groups, such as medical assistants, therapists, and administrative staff, have also been shown to experience workplace violence, often with significant mental health consequences, including burnout [5–7]. For instance, in a study conducted in Wuhan, China, two years after the COVID-19 outbreak, medical assistants and therapists were among the healthcare staff surveyed, with 31.5% overall reporting workplace violence and 37.3% showing signs of burnout [5].

Despite the breadth of this research, certain frontline professions remain underrepresented in empirical studies. Notably, emergency medical service (EMS) workers, who are often the first responders in highly volatile environments, are frequently grouped with other health care workers, masking their specific risk profile. Although violence in the EMS has been discussed since 1978 [8], it was not the focus of the large reviews mentioned above. Addressing this gap, a separate systematic review focusing on EMS workers revealed that between 57% and 93% of workers experienced verbal or physical violence, most frequently verbal abuse, physical assault, or intimidation [9].

Recent studies confirm that workplace violence against health care workers has a significant impact on burnout [9, 10]. Since burnout is a condition that can lead employees to leave work for indefinite lengths of time, up to long-term resignation and loss of the ability to work, it has been described as one of the most important occupational health problems [11]. Violence, and by extension burnout, could jeopardize the maintenance of a healthy and long-term sustainable workforce and its performance. This is particularly critical as healthcare systems already face significant challenges, including workforce shortages and demographic shifts. The COVID-19 pandemic has, among other issues, such as workload pressure and skill shortages [12], additionally contributed to an increase in burnout among health care workers [13, 14]. It has also been linked to an increase in violent incidents involving physicians, nurses, and other health care professionals, including EMS workers [14, 15]. Nurses [16, 17] and EMS workers [9], who already face an elevated baseline risk of burnout, are particularly vulnerable, as they are regularly confronted with verbal and/or physical violence in their daily work.

The attractiveness of the nursing and EMS profession has decreased, but the need for skilled workers has increased in recent decades, as reflected in the increasing shortage of skilled workers [18], partly caused by the growing demand for healthcare, primarily resulting from demographic change and an ageing population.

A review of German studies identified four papers on violence in emergency medicine, some of which reported that verbal and physical violence may be experienced by almost every participant at least once in 12 months [19]. A systematic review on health problems or violence experiences of nurses in different health care settings in Germany identified 14 studies on nurses working in acute care hospitals, with three studies reporting on the violence experienced by nurses [20]. One of the studies reported a prevalence of violence of 84% for experiencing verbal aggression and 74% for experiencing physical aggression at least four times a year [21].

Given the increasing demand for healthcare workers, particularly in nursing and EMS, driven by demographic changes and an aging population, understanding how violence and burnout interact in these high-risk groups is crucial for informing retention strategies and occupational health interventions. In addition, there is still a need for further research on workplace violence experienced by nurses and EMS workers, as the number of available studies remains limited. Therefore, this study addresses a critical research gap by focusing on nurses and EMS personnel as two frontline professions with high vulnerability but differing work environments. While both are likely to be exposed to workplace violence, the frequency, type, and psychological impact may vary. We hypothesize that workplace violence is associated with an increased risk of burnout in both professional groups. However, differences between these two professions regarding experiences of physical and psychological workplace violence, as well as burnout levels, may exist [22]. Therefore, this study aimed to investigate workplace violence and its association with the risk of burnout among nurses and EMS workers in Germany.

## Materials and methods

### Study design and participants

This cross-sectional study was conducted in Germany among professional nurses in acute care hospitals and among EMS workers in 2015. For nurses, the G-NWI (German Nursing Work Index) follow-up study of the Registered Nurse Forecasting (RN4CAST) study was conducted from March to December in 2015. A convenience sample of 71 acute care hospitals with at least 100 beds was included. Hospitals specializing in psychiatry, rehabilitation, and childcare were excluded. Nurses working in inpatient wards were invited by their employers, who had received the surveys from the study team, to voluntarily and anonymously complete the paper questionnaire. A prepaid envelope was included to send back to the Berlin University of Technology. Further details on the original RN4CAST study can be found elsewhere [23].

EMS workers were surveyed within the “Emergency medical services in Germany” (EMSiG) project [24]. Nonphysician EMS workers, such as paramedics (with 2 and 3 years of education) and emergency medical technicians (EMTs), were acquired via social media channels (Facebook groups), German EMS journals (“RETTUNGSDIENST”, “retten!”), and the professional association for EMS workers (“Deutscher Berufsverband Rettungsdienst e.V.”). Data collection was conducted over a 6-month period from June to December 2015 using the online survey tool SoSciSurvey. As a reminder, the invitation was repeated three times on social media channels. Further details can be found elsewhere [24].

### Survey instrument

The questionnaires for both nurses and EMS workers included identical questions on burnout, experience of work violence, job satisfaction, quality of care, recommendation of the current workplace, and sociodemographic data (Additional file 1). Questions regarding education were adapted according to occupational peculiarities in professional training (Additional file 2 for details on EMS workers).

#### Outcome variables

Burnout was measured using the validated Maslach Burnout Inventory–Human Services Survey (MBI-HSS; subgroup medicine), an instrument based on 22 items that determines the risk of burnout in three dimensions: EE, DP, and reduced personal accomplishment (PA) [25]. While each statement is rated based on a seven-point Likert scale according to its frequency (range: never (1), once a year or less frequently (2), once a month or less frequently (3), several times a month (4), once a week (5), several times a week (6), daily (7)), sum scores of the items in a respective dimension reflect the risk of burnout ranging from ‘high’ to ‘low’. According to the manual, participants are assigned a high risk of burnout with a sum score of ≥27 in the EE dimension, ≥10 in the DP dimension, and ≤33 in the PA dimension. For moderate risk, it is a sum score of 19–26 in EE, 6–9 in DP, and 34–39 in PA, and for low risk, a sum score of ≤ 18 in EE, ≤ 5 in DP, and ≥ 40 in PA.

Previous studies on nonphysician health care workers [26, 27] have already reported high levels of EE and DP. Consistent with these findings, we also assumed that individuals scoring high in either of the two dimensions were experiencing symptoms of burnout. The personal accomplishment subscale was excluded from the analysis, as its conceptual validity has been questioned in previous research [28, 29]. Therefore, this study focuses exclusively on the EE and DP dimensions. The internal consistency of the MBI, determined using Cronbach’s alpha, can be described as acceptable to good for the sample of nurses (EE: α = 0.895, DP: α = 0.755, MBI all dimensions: α = 0.744) and for EMS workers (EE: α = 0.895, DP: α = 0.741, MBI all dimensions: α = 0.738).

#### Exposure variables

Workplace violence, i.e., the frequencies of experiencing complaints from patients and/or their families, verbal abuse, and physical violence by patients, their families, or staff, was measured using the same seven-point Likert scale (never to daily) as the MBI. Scale values of 1 to 3 and 4 to 7 were dichotomized to define less frequent and frequent occurrences of workplace violence in this analysis.

Nurses and EMS workers were asked about their satisfaction with their current job and their career choice using a four-point Likert scale with response options “very dissatisfied”, “rather dissatisfied”, “rather satisfied”, and “very satisfied”. Additionally, they were asked whether they would recommend their hospital or EMS organization as a good place to work with responses “no”, “rather no”, “rather yes”, and “yes”. The perceived quality of care on the ward or in the EMS setting was also assessed using a four-point Likert scale (poor, fair, good, excellent). For statistical analysis and to enhance interpretability, all variables were dichotomized. Educational categories were consolidated for EMS workers: all EMS training programs shorter than 2 years were grouped under the category EMT.

**Fig. 1:**
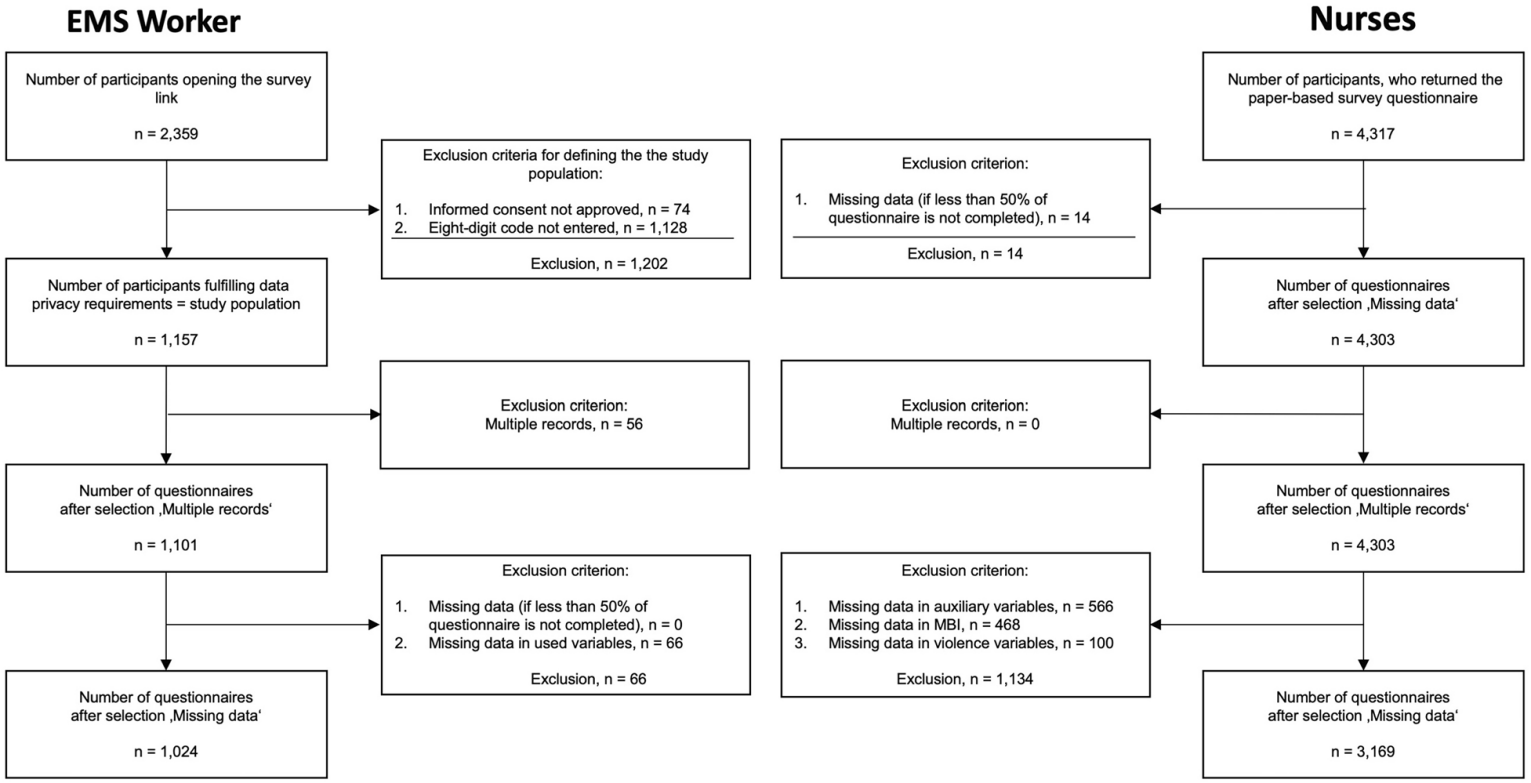
Flowchart of participant selection

We applied a complete case analysis approach for both the EMS worker and the nurse samples. All records were checked for plausibility and completeness. The exclusion of nurses’ survey data resulted from missing data (auxiliary variables *n* = 566, MBI *n* = 468, violence variables *n* = 100, ward numbers = 14). A total of *n* = 3,169 observations of nurse participants (73.4% out of *n* = 4,317) were included in the analysis (Fig. 1).

For EMS workers, failure to confirm the mandatory privacy statement (*n* = 74) or the eight-digit personalized code (*n* = 1128), which was necessary to ensure that the data record could be deleted retroactively at the request of the participant, led to the exclusion of the respective survey data. Further data were excluded due to multiple records (*n* = 56) or missing data (*n* = 77). An EMS worker sample of *n* = 1,024 observations was included in the analysis (43.4% out of *n* = 2359) (Fig. 1).

### Statistical analysis

We applied complete case analysis (CCA) to both the nurse and EMS worker samples. Observations with missing values on key variables (e.g., burnout dimensions, exposure to workplace violence, sociodemographic characteristics) were excluded from the regression models. We assessed the distribution of missing values across all variables and conducted bias checks by comparing complete and incomplete cases. For categorical variables, χ^2^-tests were used; for the continuous variable work experience in years, we applied the Mann–Whitney U test. Statistical significance was determined using a threshold of *p* < 0.05. We followed established recommendations for evaluating the representativeness of complete-case samples [30, 31].

A descriptive analysis was conducted to examine sociodemographic characteristics and the level of burnout risk, following the guideline of the MBI manual [25]. All descriptive analyses were performed separately for nurses and EMS workers and were stratified by sex. In addition, we applied the Chi-squared test (χ2) to test for associations between gender and burnout level.

Spearman correlation coefficients were calculated to assess the relationship between the continuous sum scores of EE and DP, separately for nurses and EMS workers. Binary logistic regression analyses were applied to investigate the associations between complaints, verbal abuse, physical violence, and a high risk of burnout in the EE and DP dimensions, stratified by nurses and EMS workers. The dependent variable was coded as 1 to indicate a high risk of burnout and 0 for low or moderate risk. Model requirements (no outliers, no multicollinearity) were assessed prior to estimation. Given the statistically significant Spearman correlation between age and work experience (nurses: 0.859; *p* < 0.001/EMS: 0.858; *p* < 0.001), age was excluded from the analyses to avoid multicollinearity. Confounders were selected based on factors that have been repeatedly associated with burnout in previous studies (e.g., demographic characteristics [32], level of qualification [33]) or that are theoretically grounded in models such as Maslach’s Burnout Framework, which highlights the relevance of workplace conditions [34]. Sociodemographic variables (e.g., gender, education), complaints, work experience, job satisfaction, and perceived quality of care were included in the logistic model using the enter method. Outliers were assessed using the 1.5−3.0 interquartile range (IQR), resulting in no exclusions.

All statistical tests were performed with a significance level set at *p* < 0.05. Analyses were conducted using IBM SPSS Statistics, Version 29.

## Results

### Comparison of complete and incomplete cases

For EMS workers, the overall proportion of missing data was low (6.99%), with no independent variable exceeding 5% missingness (range 0–4.4%). A single statistically significant difference between complete and incomplete cases was observed only for complaints from patients and/or their families (χ^2^ = 6.66, *p* = 0.010). Given the very small proportion of missing cases for this variable (1,3%), and the fact that it is not the sole focus of the analysis but rather one of several variables examined, any resulting bias in regression estimates is likely to be negligible. For nurses, the overall proportion of missing data was higher (26.27%). Only work experience in years (5.6%) and MBI EE (6.4%) exceeded 5% missingness, and neither showed significant differences between complete and incomplete cases. Significant differences were observed for physical violence by patients and/or their families (χ^2^ = 5.241, *p* = 0.022), gender (χ^2^ = 19.562, *p* < 0.001), and MBI DP (χ^2^ = 13.549, *p* < 0.001). Although the proportion of missing cases for physical violence, gender, and MBI DP was small (1.3%, 1.7%, and 3.8%, respectively), outcome-related missingness for MBI DP could introduce some bias, which is acknowledged as a limitation. Potential bias from the imbalance in gender distribution was mitigated by including gender as a covariate in all regression models, thereby accounting for its influence on the associations of interest.

Given that (a) the extent of missing data was low for EMS workers and moderate for nurses; (b) most variables showed no significant differences between complete and incomplete cases; and (c) the few significant differences were of small magnitude or involved covariates adjusted for in the models, we considered CCA an appropriate approach. This method preserves interpretability and avoids the complexities of multiple imputations, while acknowledging that a small degree of bias cannot be entirely excluded.

### Participant characteristics and their experiences of workplace violence

The demographic data show that the gender distribution in nurses, with a share of female employees at 84.7%, is almost opposite to that in EMS workers, where 86.4% of the employees are male (Table 1). Most nurses were between 45 and 54 years old (28.6%), worked full-time (63.2%), and had an average of 16.9 years of work experience. In comparison, most EMS workers were between 25 and 34 years old (38.1%), worked full-time (89.7%), and had an average of 12.2 years of work experience.

**Table 1:** Descriptive statistics on participants and their experiences of workplace violence

	Nurses			Emergency medical services (EMS) workers		
	Female	Male	Total	Female	Male	Total
Gender in % (n)	84.7 (*n* = 2685)	15.3 (*n* = 484)	100 (*n* = 3169)	13.6 (*n* = 139)	86.4 (*n* = 885)	100 (*n* = 1024)
Age group in %						
≤ 24	10.8	8.9	10.6	35.3	17.4	19.8
25–34	27.3	32.7	28.1	48.2	36.5	38.1
35–44	20.5	25.8	21.3	12.2	29.0	26.8
45–54	29.1	26.2	28.6	3.6	14.4	12.9
≥ 55	12.3	6.4	11.4	0.7	2.7	2.4
Full-time in %	60.0	81.4	63.2	87.1	90.2	89.7
Work experience in years: mean (SD)	17.5 (11.9)	13.7 (9.6)	16.9 (11.7)	7.4 (6.0)	12.9 (9.1)	12.2 (9.0)
Level of qualification in %						
Diploma nurse (3-year vocational training)	97.0	96.5	96.9	n.a	n.a	n.a
Degree in nursing (bachelor or master)	3.0	3.5	3.1	n.a	n.a	n.a
Paramedic (3-year education)	n.a	n.a	n.a	4.3	12.4	11.3
Paramedic (2-year education)	n.a	n.a	n.a	79.9	73.1	74.0
Emergency medical technician (EMT)	n.a	n.a	n.a	15.8	14.5	14.7
Working conditions in %						
Very/rather dissatisfied with the choice of the career	30.6	34.5	31.2	35.3	28.7	29.6
Very/rather dissatisfied with the current job	45.8	47.1	46.0	43.9	45.6	45.4
Definitely/probably no recommendation of the current workplace as a good place to work	42.7	42.6	42.7	48.9	54.4	53.6
Poor to fair quality of care	41.6	44.6	42.1	25.2	30.8	30.1
Occurrence of workplace violence, daily to several times a month in %						
Complaints from patients and/or their families	45.5	48.1	45.9	13.7	15.0	14.8
Verbal abuse by patients and/or their families	44.3	46.7	44.7	61.2	59.7	59.9
Verbal abuse by staff	18.1	19.8	18.4	20.1	16.7	17.2
Physical violence by patients and/or their families	12.0	13.0	12.2	23.7	23.5	23.5
Physical violence by staff	0.3	0.6	0.4	1.4	0.7	0.8

Most nurses had completed a 3-year vocational training in nursing; only 3.1% held a bachelor’s or master’s degree. In the EMS, 74.0% of participants had 2 years of paramedic education (“Rettungsassistent”), 11.3% had 3 years of paramedic education (“Notfallsanitäter”), and 14.7% were trained as emergency medical technicians. Both nurses and EMS workers tended to be dissatisfied, with more EMS workers (53.6%) not recommending their field of work as a good place to work compared to nurses (42.7%). A higher percentage of nurses (42.1%) compared to EMS workers (30.1%) rated the quality of care as poor to fair.

A threefold higher percentage of female (45.5%) and male nurses (48.1%) reported receiving complaints from patients and/or their families on a frequent (i.e., daily to several times a month) basis compared to female (13.7%) and male EMS workers (15.0%) (Table 1). The proportion of EMS workers who reported experiencing physical violence from patients and/or their families with similar frequency was almost twice as high (23.7% female and 23.5% male) as that of nurses (12.0% female and 13.0% male). Furthermore, verbal abuse by patients and/or their families was more frequently reported by EMS workers than by nurses (59.9% versus 44.7%), with more than 15% variation for female staff (EMS 61.2% versus 44.3%). Verbal abuse by staff was experienced frequently by a comparable share of both professional groups (EMS: 20.1% female/16.7% male versus nurses: 18.1% female/19.8% male).

### Descriptive results on the risk of burnout

Spearman correlation coefficients were calculated between the continuous sum scores of EE and DP, separately for nurses and EMS workers. The results indicated a statistically significant positive association for both nurses (ρ = 0.401, *p* < 0.01) and EMS workers (ρ = 0.368, *p* < 0.01), suggesting that the two burnout dimensions are related but still distinct.

In a separate step, burnout risk was classified as low, moderate, or high based on threshold values provided in the MBI manual (3rd Edition) for the EE and DP dimensions (see section ‘Survey instruments’) [25]. The results were further stratified by gender and are presented in Table 2 as both percentages and absolute response counts.

**Table 2:** Risk of burnout among nurses and EMS workers by gender

	Nurses			Emergency medical services (EMS) workers		
Maslach Burnout Inventory (MBI) dimensions	Female (*n* = 2685)	Male (*n* = 484)	Total (*n* = 3169)	Female (*n* = 139)	Male (*n* = 885)	Total (*n* = 1024)
Emotional exhaustion (EE)						
High risk of burnout in %	23.8	26.4	24.2	26.6	25.6	25.8
Moderate risk of burnout in %	34.5	31.0	33.9	30.2	30.5	30.5
Low risk of burnout in %	41.7	42.6	41.8	43.2	43.8	43.8
Sum in %	∑ = 100	∑ = 100	∑ = 100	∑ = 100	∑ = 100	∑ = 100
Depersonalization (DP)						
High risk of burnout in %	29.9	44.2	32.1	33.8	40.5	39.6
Moderate risk of burnout in %	26.3	26.2	26.3	36.7	34.5	34.8
Low risk of burnout in %	43.8	29.5	41.7	29.5	25.1	25.7
Sum in %	∑ = 100	∑ = 100	∑ = 100	∑ = 100	∑ = 100	∑ = 100

Overall, nurses and EMS workers showed a higher risk of burnout in the DP dimension than in the EE dimension, with EMS workers exhibiting even higher DP values (39.6%) compared to nurses (32.1%) (Table 2). The proportion of participants at high risk of burnout in the DP dimension was higher for males than for females in both professions for both EMS workers (+ 6.7%) and nurses (+ 14.3%). Conversely, the proportion at low risk was higher among females compared to males (EMS workers: + 4.4%; nurses: + 14.3%). Regarding the EE dimension, there were only small differences between the professions and between males and females.

A significant association between gender and risk of burnout was found for the DP dimension among nurses (χ2 = 46.428; *p* < 0.001, df = 2). In contrast, no significant associations were observed for the EE dimension in either profession, nor for the DP dimension among EMS workers (DP EMS: χ2 = 2.421, *p* = 0.298, df = 2; EE nurses: χ2 = 2.670; *p* = 0.263, df = 2; EE EMS: χ2 = 0.06; *p* = 0.971, df = 2).

### Association between workplace violence and the risk of burnout

The associations between a high risk of burnout in the EE and DP dimensions and exposure to work violence were analyzed for both professions. Adjusted odds ratios (ORs) were reported to estimate the strength and direction of the association between specific exposures and the likelihood of being at high risk for burnout, while controlling for potential confounding variables. An OR > 1 indicates increased odds compared to the reference group. The reference groups for all regression models are specified in Table 3.

**Table 3:** Binary logistic regression analyses: association between burnout and workplace violence

Variables	High risk of burnout (emotional exhaustion, EE)				High risk of burnout (depersonalization, DP)			
	Nurses		Emergency medical services (EMS) workers		**Nurses**		**Emergency medical services (EMS) workers**	
	Odds ratio	95% CI	Odds ratio	95% CI	**Odds ratio**	**95% CI**	**Odds ratio**	**95% CI**
Workplace violence (daily to several times a month)								
Complaints from patients or their families	1.56***	1.26–1.94	1.17	0.76–1.80	1.72***	1.43–2.07	1.24	0.83–1.85
Verbal abuse by patients and/or families	1.37**	1.10–1.72	1.01	0.69–1.47	1.57***	1.29–1.90	1.56**	1.16–2.08
Verbal abuse by staff	1.24	0.99–1.56	1.39	0.92–2.08	1.20	0.97–1.48	2.25***	1.51–3.35
Physical violence by patients and/or families	1.52**	1.16–2.00	1.60*	1.07–2.38	1.47**	1.15–1.89	1.26	0.88–1.79
Physical violence by staff	1.30	0.32–5.35	2.82	0.59–13.53	0.84	0.24–2.98	2.59	0.29–23.24
Constant	0.02***		0.03***		0.17***		0.45*	
Demographics								
Gender (male)	1.03	0.79–1.33	0.82	0.51–1.32	1.67***	1.34–2.07	1.35	0.91–2.00
Employment (full-time)	1.73***	1.40–2.13	1.36	0.77–2.38	1.30**	1.08–1.56	1.35	0.87–2.09
Work experience in years	1.02***	1.01–1.03	1.02*	1.00–1.04	0.97***	0.96–0.98	0.97***	0.96-0.99
Level of education								
Degree in nursing (bachelor or master)	1.47	0.88–2.46			1.29	0.82–2.02		
Paramedic (3-year education)			1.09	0.54–2.18			0.85	0.50–1.46
Paramedic (2-year education)			1.50	0.90–2.49			1.05	0.71–1.55
Working conditions								
Very/a little dissatisfied with the current job	2.81***	2.22–3.54	3.70***	2.56–5.36	1.37**	1.11–1.68	0.87	0.64–1.19
Very/a little dissatisfied with the choice of the career	2.45***	2.03–2.97	1.91***	1.37–2.67	1.78***	1.49–2.12	1.52**	1.12–2.07
Definitely no or probably no recommendation of the current work-area as a good place to work	2.07***	1.66–2.59	2.15***	1.45–3.17	1.28*	1.04–1.56	1.57**	1.16–2.13
Poor to fair quality of care	1.35**	1.10–1.65	1.63**	1.16–2.28	1.46***	1.22–1.74	1.51**	1.11–2.06
R^2^	.31		0.30		0.21		0.14	

^***^ *p* < 0.001; ** *p* < 0.01; * *p* < 0.05

Reference groups:

Workplace violence: never to once a month or less

Demographics: female (gender); part-time, temporary or voluntary work employment status

Level of education: nurses with 3-year vocational education, respectively, emergency medical technician (EMT)

Working conditions: very or a little satisfied with the current job and choice of the career; definitely or probably recommending the current work area as a good place to work; good to excellent quality of care

Nurses had a significantly higher odds of risk of burnout in both the EE and DP dimensions if they had experienced workplace violence in the form of daily to several times a month from patients or their families (EE: OR 1.56; 95% CI 1.26–1.94 and DP: OR 1.72; 95% CI 1.43–2.07). Similarly, verbal abuse by patients and/or families was associated with increased odds (EE: OR 1.37; 95% CI 1.10–1.72 and DP: OR 1.57; 95% CI 1.35–2.19), as was physical violence (EE: OR 1.52; 95% CI 1.16–2.00 and DP: OR 1.47; 95% CI 1.15–1.96) (Table 3).

In comparison, EMS workers had significantly higher odds of being at high risk of burnout in the EE dimension when having experienced physical violence (OR 1.60; 95% CI 1.07–2.38) and in the DP dimension when having experienced verbal abuse by patients and/or their families (OR 1.56; 95% CI 1.16–2.08). Additionally, frequent verbal abuse by staff was associated with higher odds of a high risk of burnout in the DP dimension for EMS workers (OR 2.25; 95% CI 1.51–3.35).

Higher odds of being at high risk of burnout were estimated for nurses who were dissatisfied with their current job (EE: OR 2.81; 95% CI 2.22–3.54 and DP: OR 1.37; 95% CI 1.11–1.68). Among EMS workers, the odds of high risk of burnout in the EE dimension were even higher (OR: 3.70; 95% CI 2.56–5.36). Furthermore, dissatisfaction with career choice, not recommending the current workplace as a good place to work, and perceiving the quality of care as poor or fair were significantly associated with increased odds of being at high risk of burnout in both burnout dimensions.

In both professions, longer work experience was significantly associated with increased odds of being at high risk of burnout in the EE dimension, while it was associated with lower odds of a high risk in the DP dimension. Full-time employment significantly increased the odds of high burnout risk only for nurses (EE: OR 1.73; 95% CI 1.40–2.13, DP: OR 1.30; 95% CI 1.08–1.56). Regarding gender, only male nurses had significantly higher odds of being at high risk of burnout in the DP dimension (OR: 1.67; 95% CI 1.34–2.07). The level of education was not significantly associated with burnout risk in any professional group or burnout dimension.

## Discussion

Compared to nurses (44.7%), a higher proportion of EMS workers (59.9%) reported experiencing verbal abuse by patients and/or their families. Additionally, 12.2% (*n* = 386) of all nurses and 23.5% (*n* = 241) of all EMS workers reported regularly experiencing physical violence from patients and/or their families. One possible explanation is that EMS workers are more frequently exposed to physical violence due to the public or domestic environment in which they provide care. Moreover, physical violence is not uncommon as a reason for dispatching EMS workers.

Burnout risk as measured by the MBI was higher in the DP dimension (nurses: 32.1%; EMS workers: 39.6%), also referred to as cynicism in newer versions of the MBI, compared to the EE dimension (nurses: 24.2%; EMS workers: 25.8%). These findings are consistent with previous research [34]. One possible explanation is that nurses are frequently exposed to intense psychological and emotional demands, likely due to the close and long-term nature of patient care in this profession. EMS workers may be more prone to emotional detachment or to perceiving patients in a rather impersonal manner. This could be attributed to shorter patient contact times, the acute and often stressful nature of emergency situations, or the absence of follow-up care. In addition, we found that a high risk of burnout in the DP dimension was more prevalent among males (nurses 44.2% / EMS 40.5%) than among females (nurses 29.9%/EMS 33.8%), a pattern also observed in previous studies [36, 37].

The regression results indicate that workplace violence and job dissatisfaction are critical health care issues. An association between physical and psychological violence and burnout has already been shown in previous literature [38]. A systematic review from Denmark in 2020 indicated associations between workplace violence and mental health problems [39]. For paramedics, verbal violence has already been identified as a significant factor associated with burnout [40]. In line with previous literature [41], our study also showed that job dissatisfaction is associated with increased odds of high risk of burnout.

For the nursing profession, further significant associations for the risk of burnout were identified in the EE dimension. These include aspects of violence, such as complaints from patients and/or families (EE: OR 1.56; 95% CI 1.26–1.94) and verbal abuse by patients and/or families (EE: OR 1.37; 95% CI 1.10–1.72). In the DP dimension, significant associations for nurses could be shown between a high risk of burnout and nearly all variables except the level of education and physical violence by staff. Recent literature shows that there is an effect between the experience of violence and depersonalization, which supports our findings [42].

This study emphasizes the importance of policies and organizational measures for the appropriate prevention and management of workplace violence, occupational stress, and burnout. The 75th World Health Assembly (2022) called on Member States to fulfill their duty of care by using the Global Health and Care Worker Compact of the World Health Organization (WHO), which includes complementary management and policy actions structured around four domains: (1) preventing harm from dangers and hazards at work, including protection from violence and harassment; (2) providing support; (3) promoting the inclusivity of rights; and (4) strengthening safeguarding rights [43].

### Practical implications

Multicomponent interventions are seen as the most effective approach to impact rates of workplace violence. To support and strengthen employees in dealing with workplace violence, preventative training in dealing with violence should be implemented [44]. Efforts should be made to improve working conditions in both professions to limit the risk of burnout from the outset and ensure patient safety. Systemic measures such as the legal prosecution of individuals who misuse emergency hotlines, the introduction of incentives to refer non-urgent patients to care providers outside of emergency departments, and improvements in dispatch protocols to better differentiate between urgent and non-urgent cases could positively impact the working conditions of EMS workers [45]. To prevent violence, e.g., in the emergency department, a comprehensive guide has been developed that recommends measures such as organizational (e.g., to handle overcrowding), technical (e.g., alarm systems), architectural (e.g., waiting areas), staff-related (e.g., de-escalation training), and patient-related interventions (e.g., education, banning) [46]. In terms of working climate, a culture of transparency, professionalism, and patient-oriented discourse among health care workers and supervisors needs to be fostered. Direct measures such as emergency call buttons for health care workers and the implementation of security protocols seem sensible.

Although the data were collected in 2015, they provide important insights into the situation at that time and offer a valuable basis for comparison with future developments. Continuously collected data would support a better understanding of the risk of burnout and violence against nursing staff and employees in the emergency services. This, in turn, will allow for more targeted evaluations and the development of more effective measures for prevention and intervention. Further research is needed to determine whether cross-occupational findings on burnout risk can be replicated between nurses and EMS workers. This question should be examined using longitudinal study designs. Comparative effectiveness research on implemented measures can then provide information on which investments yield the greatest benefits.

### Limitations

The following limitations should be mentioned in connection with the interpretation of the studies' results: (1) unmeasured confounding factors at the individual, hospital, and community levels may influence the results and bias estimates. (2) Due to the cross-sectional study design, causal conclusions are not possible. Additionally, it cannot be ruled out that some responses may contain inaccurate or incorrect information or that the high number of mandatory fields in the EMS survey due to the obligation to respond has led to an increased dropout rate. The dropout rate refers to participants who did not provide informed consent or failed to enter a required personalized code. (3) The exclusion of rehabilitation, childcare, and especially psychiatric nurses may underestimate the risk of burnout. (4) The data collected from EMS workers via an online survey may be subject to selection bias due to limited accessibility of potential participants as being older and female have been associated with lower internet use [47]. A further limitation is the lack of a response rate for EMS workers, as participants were recruited via EMS journals, social media, and a professional association to participate in an online survey. As satisfaction measures are based exclusively on the personal subjective assessments of the study participants, satisfaction and a high risk of burnout may be over- or underestimated [48, 49]. (5) The regression analysis was based on complete cases. We acknowledge that CCA assumes that data are missing completely at random (MCAR) or that deviations from MCAR are minimal. While our bias checks suggest that substantial violations of this assumption are unlikely, the significant difference for MBI DP warrants caution. As an outcome variable, even small amounts of missingness in MBI DP could lead to minor distortions in regression estimates. Therefore, findings for nurses focusing on the DP dimension should be interpreted with caution, particularly regarding variables with significant differences between complete and incomplete cases. Nevertheless, for gender, the difference likely reflects the overall gender imbalance within the nursing profession rather than systematic bias introduced through case exclusion.

## Conclusion

This is the first large-scale German study to analyze workplace violence and the risk of burnout among nurses and EMS workers. The results reveal a high prevalence of both verbal and physical violence, particularly among EMS workers, and significant associations between these experiences and a high risk of burnout. These findings are in line with existing literature and underscore the urgent need for preventive and supportive measures. In addition to violence, factors such as job dissatisfaction and perceived poor to fair quality of care were strongly associated with burnout risk, especially in the dimensions of EE and DP. To address these issues, efforts should focus on improving working conditions, establishing support systems for affected staff, and implementing targeted violence prevention strategies. Further longitudinal research is needed to investigate causal pathways and to evaluate effective interventions across healthcare professions.

## Supplementary Information

Additional file 1.

Additional file 2.

## Data Availability

The data gathered and analyzed during the study can be made available from the corresponding author upon reasonable request.

## Appendix A

See Table 4

**Table 4:** Binary logistic regression analyses: unadjusted association between burnout and workplace violence

	High risk of burnout (emotional exhaustion, EE)				High risk of burnout (depersonalization, DP)			
	Nurses		Emergency medical services (EMS) workers		Nurses		Emergency medical services (EMS) workers	
Variables	Unadjusted OR	95% CI	Unadjusted OR	95% CI	Unadjusted OR	95% CI	Unadjusted OR	95% CI
Workplace violence (daily to several times a month)								
Complaints from patients or their families	2.70***	2.28–3.19	1.74**	1.20–2.51	2.91***	2.50–3.40	1.90***	1.31–2.75
Verbal abuse by patients and/or families	2.75***	2.33–3.26	1.48**	1.10–1.98	2.84***	2.44–3.32	2.02***	1.57–2.61
Verbal abuse by staff	2.55***	2.11–3.09	2.23***	1.58–3.13	2.17***	1.80–2.60	2.93***	2.01–4.26
Physical violence by patients and/or families	2.53***	2.03–3.16	2.19***	1.61–2.99	2.72***	2.19–3.38	2.03***	1.49–2.77
Physical violence by staff	3.14*	1.01–9.77	4.87*	1.16–20.53	2.13	0.68–6.61	5.33	0.65–43.45
Demographics								
Gender (male)	1.15	0.92–1.43	0.95	0.63–1.43	1.86***	1.53–2.27	1.12	0.78–1.60
Employment (full-time)	1.34***	1.12–1.59	1.35	0.82–2.21	1.64***	1.39–1.92	1.34	0.90–2.01
Work experience in years	1.01*	1.00–1.02	1.02**	1.01–1.04	0.97***	0.96–0.97	0.98***	0.96–0.99
Level of education								
Degree in nursing (bachelor or master)	1.13	0.72–1.79			1.30	0.86–1.96		
Paramedic (3-year education)			1.27	0.70–2.29			0.69	0.42–1.12
Paramedic (2-year education)			1.58*	1.02–2.44			0.96	0.65–1.33
Working conditions								
Very/a little dissatisfied with the current job	6.26***	5.18–7.56	6.68***	4.82–9.26	2.33***	2.00–2.72	1.40**	1.09–1.80
Very/a little dissatisfied with the choice of the career	3.63***	3.06–4.30	3.01***	2.24–4.04	2.29***	1.96–2.68	1.70***	1.29–2.25
Definitely no or probably no recommendation of the current work-area as a good place to work	5.16***	4.31–6.17	5.12***	3.66–7.16	2.10***	1.80–2.44	1.87***	1.46–2.40
Poor to fair quality of care	2.95***	2.50–3.49	2.59***	1.94–3.48	2.27***	1.95–2.64	1.92***	1.45–2.54

****p* < 0.001; *** p* < 0.01; * *p* < 0.05

Reference groups:

Workplace violence: never to once a month or less

Demographics: female (gender); part-time, temporary or voluntary work employment status

Level of education: nurses with 3-year vocational education, respectively

Emergency medical technician (EMT)

Working conditions: very or a little satisfied with the current job and choice of the career; definitely or probably recommending the current work area as a good place to work; good to excellent quality of care

## Appendix B

The G-NWI (German Nursing Work Index) study was a follow-up of the RN4Cast study in Germany, which was conducted using the same questionnaire. The RN4Cast study was a cross-national, multistage, cross-sectional study designed to obtain important unmeasured factors in prediction models, including how characteristics of the hospital work environment affect nurse recruitment, retention and patient outcomes. The study was conducted in 12 participating European countries with at least 30 general acute care hospitals. A detailed description of the study can be found in Sermeus et al. Nurse forecasting in Europe (RN4CAST): Rationale, design, and methodology. For the follow-up survey of the RN4CAST study, a total of 4,317 nurses in 71 acute care hospitals were recruited for the survey. The acute-care hospitals were recruited by email in 2014 (email addresses from the German Hospital Directory, White List, GBA). Mainly normal wards (primarily surgical and internal medicine wards) were included in the study. One contact person per hospital distributed the questionnaires to selected wards. The return envelopes were enclosed directly. All participating hospitals received regular feedback on the return of the questionnaires.

The EMSiG project is dedicated to the EMS in Germany, which has not yet been systematically investigated in research. The aim of the project is to systematize the heterogeneous provision of EMS and to investigate the effectiveness, cost-effectiveness, and efficiency of this sector, as well as to determine preferences for EMS.

## Abbreviations

DP: Depersonalization
EE: Emotional exhaustion
EMS: Emergency medical services
EMS-SAQ: Emergency medical services - Safety Attitudes Questionnaire
EMT: Emergency medical technician (EMT-I-intermediate, EMT-B-basic)
MBI: Maslach Burnout Inventory
MBI-HSS: Maslach Burnout Inventory–Human Services Survey
PA: Personal accomplishment
RN4CAST: Nurse Forecasting in Europe
SD: Standard deviation

## References

[1] Li Y-L, Li R-Q, Qiu D, Xiao S-Y. Prevalence of workplace physical violence against health care professionals by patients and visitors: a systematic review and meta-analysis. Int J Environ Res Public Health. 2020;17:299. 31906306 10.3390/ijerph17010299 PMC6982349

[2] Wiskow C. Guidelines on Workplace Violence in the Health Sector. Comparison of major known national guidelines and strategies: United Kingdom, Australia, Sweden, USA (OSHA and California). Geneva, Switzerland; 2003.

[3] International Labour Office (ILO) International Council of Nurses (ICN) World Health Organization (WHO) Public Services International (PSI). Framework Guidelines for Addressing Workplace Violence in the Health Sector.. Geneva, Switzerland: ILO; 2002 [cited 2025 Apr 12]. p. 31. https://iris.who.int/bitstream/handle/10665/42617/9221134466.pdf?sequence=1&isAllowed=y

[4] Liu J, Gan Y, Jiang H, Li L, Dwyer R, Lu K, et al. Prevalence of workplace violence against healthcare workers: a systematic review and meta-analysis. Occup Environ Med. 2019;76:927–37. 10.1136/oemed-2019-105849. 31611310 10.1136/oemed-2019-105849

[5] Wang F, Zhang M, Xiong N, Huang Y, Tang Y, He C, et al. Workplace violence and burnout among health workers two years after the COVID-19 outbreak in Wuhan, China: the chain mediation effect of sleep disturbance and work ability. Healthcare. 2024;12:1903. 39337244 10.3390/healthcare12181903 PMC11431534

[6] Cascales-Martínez A, López-Ros P, Pina D, Cánovas-Pallares JM, López López R, Puente-López E, et al. Differences in workplace violence and health variables among professionals in a hospital emergency department: a descriptive-comparative study. PLoS ONE. 2024;19:e0314932. 10.1371/journal.pone.0314932. 39636963 10.1371/journal.pone.0314932 PMC11620588

[7] Alhomoud F. ‘That’s Enough’ - Workplace Violence Against Physicians, Pharmacists, and Nurses in Saudi Arabia: A Systematic Review of Prevalence, Causes, and Consequences. Risk Manag Healthc Policy. 2025;Volume 18:373–408. https://www.dovepress.com/thats-enough---workplace-violence-against-physicians-pharmacists-and-n-peer-reviewed-fulltext-article-RMHP 10.2147/RMHP.S509895 PMC11995410 40230661

[8] Sutherland A, Strang L, Stepanek M, Giacomantonio C, Boyle A. Using Ambulance Data for Violence Prevention. Santa Monica, CA; 2017. https://www.rand.org/pubs/research_reports/RR2216.html

[9] Murray RM, Davis AL, Shepler LJ, Moore-Merrell L, Troup WJ, Allen JA, et al. A systematic review of workplace violence against emergency medical services responders. New Solut. 2020;29:487–503. 10.1177/1048291119893388. 31841060 10.1177/1048291119893388 PMC8594050

[10] Vincent-Höper S, Stein M, Nienhaus A, Schablon A. Workplace aggression and burnout in nursing—the moderating role of follow-up counseling. Int J Environ Res Public Health. 2020;17:3152. 32369903 10.3390/ijerph17093152 PMC7246829

[11] Gómez-Urquiza JL, Fuente-Solana EI, Albendín-García L, Vargas-Pecino C, Ortega-Campos EM, Fuente GA. Prevalence of burnout syndrome in emergency nurses: a meta-analysis. Crit Care Nurse. 2017;37:e1-9. 10.4037/ccn2017508. 28966203 10.4037/ccn2017508

[12] Lim MC, Jeffree MS, Saupin SS, Giloi N, Lukman KA. Workplace violence in healthcare settings: The risk factors, implications and collaborative preventive measures. Annals of Medicine & Surgery. 2022;78. 10.1016/j.amsu.2022.103727 10.1016/j.amsu.2022.103727 PMC9206999 35734684

[13] Salazar-de-Pablo G, Vaquerizo-Serrano J, Catalan A, Arango C, Moreno C, Ferre F, et al. Impact of coronavirus syndromes on physical and mental health of health care workers: systematic review and meta-analysis. J Affect Disord. 2020;275:48–57. 32658823 10.1016/j.jad.2020.06.022 PMC7314697

[14] McKay D, Heisler M, Mishori R, Catton H, Kloiber O. Attacks against health-care personnel must stop, especially as the world fights COVID-19. Lancet. 2020;395:1743–5. 32445692 10.1016/S0140-6736(20)31191-0 PMC7239629

[15] Deutsches Ärzteblatt. Gewalt gegen Gesundheitspersonal auch in der Coronakrise: Weltärztebund ruft zum Handeln auf. [cited 2021 Jan 24]. https://www.aerzteblatt.de/nachrichten/113057/Gewalt-gegen-Gesundheitspersonal-auch-in-der-Coronakrise-Weltaerztebund-ruft-zum-Handeln-auf

[16] Blackstock S, Salami B, Cummings GG. Organisational antecedents, policy and horizontal violence among nurses: an integrative review. J Nurs Manag. 2018;26:972–91. 10.1111/jonm.12623. 30171643 10.1111/jonm.12623

[17] Edward K, Stephenson J, Ousey K, Lui S, Warelow P, Giandinoto J. A systematic review and meta-analysis of factors that relate to aggression perpetrated against nurses by patients/relatives or staff. J Clin Nurs. 2016;25:289–99. 10.1111/jocn.13019. 26507792 10.1111/jocn.13019

[18] Bundesministerium für Gesundheit. Beschäftigte in der Pflege. 2018 [cited 2021 Jan 24]. https://www.bundesgesundheitsministerium.de/index.php?id=646

[19] Hofmann T, Hachenberg T. Gewalt in der Notfallmedizin–gegenwärtiger Stand in Deutschland. AINS - Anästhesiologie · Intensivmedizin · Notfallmedizin · Schmerztherapie. 2019;54:146–54. 10.1055/s-0043-112189. 10.1055/s-0043-112189 30769354

[20] Schaller A, Klas T, Gernert M, Steinbeißer K. Health problems and violence experiences of nurses working in acute care hospitals, long-term care facilities, and home-based long-term care in Germany: a systematic review. PLoS ONE. 2021;16:e0260050. 10.1371/journal.pone.0260050. 34793537 10.1371/journal.pone.0260050 PMC8601565

[21] Raspe M, Koch P, Zilezinski M, Schulte K, Bitzinger D, Gaiser U, et al. Arbeitsbedingungen und Gesundheitszustand junger Ärzte und professionell Pflegender in deutschen Krankenhäusern. Bundesgesundheitsblatt Gesundheitsforsch Gesundheitsschutz. 2020;63:113–21. 10.1007/s00103-019-03057-y. 10.1007/s00103-019-03057-y 31720739

[22] Bundesagentur für Arbeit. Beschäftigte nach Berufen (Klassifikation der Berufe 2010) - Deutschland, West/Ost und Länder (Quartalszahlen) - September 2015. 2015 [cited 2024 Jan 20]. https://statistik.arbeitsagentur.de/DE/Navigation/Statistiken/Themen-im-Fokus/Berufe/Berufe-Nav.html

[23] Sermeus W, Aiken LH, Van den Heede K, Rafferty AM, Griffiths P, Moreno-Casbas MT, et al. Nurse forecasting in Europe (RN4CAST): rationale, design and methodology. BMC Nurs. 2011;10:6. 10.1186/1472-6955-10-6. 21501487 10.1186/1472-6955-10-6 PMC3108324

[24] Baier N, Roth K, Felgner S, Henschke C. Burnout and safety outcomes - a cross-sectional nationwide survey of EMS-workers in Germany. BMC Emerg Med. 2018;18:24. 10.1186/s12873-018-0177-2. 30126358 10.1186/s12873-018-0177-2 PMC6102842

[25] Maslach C, Jackson SE, Leiter MP. Maslach burnout inventory manual - 3rd edition. 1996.

[26] Dyrbye LN, Major-Elechi B, Thapa P, Hays JT, Fraser CH, Buskirk SJ, et al. Characterization of nonphysician health care workers’ burnout and subsequent changes in work effort. JAMA Netw Open. 2021;4:e2121435. 34415312 10.1001/jamanetworkopen.2021.21435 PMC8379653

[27] West CP, Dyrbye LN, Sloan JA, Shanafelt TD. Single item measures of emotional exhaustion and depersonalization are useful for assessing burnout in medical professionals. J Gen Intern Med. 2009;24:1318–21. 19802645 10.1007/s11606-009-1129-z PMC2787943

[28] Schaufeli W, Enzmann D. The Burnout Companion to Study and Practice: A Critical Analysis. CRC Press; 2020. https://www.taylorfrancis.com/books/9781000124118

[29] Green DE, Walkey FH, Taylor AJW. The three-factor structure of the Maslach Burnout Inventory: a multicultural, multinational confirmatory study. J Soc Behav Pers. 1991;6:453–73.

[30] Schafer JL, Graham JW. Missing data: our view of the state of the art. Psychol Methods. 2002;7:147–77. 12090408

[31] Little R, Rubin D. Statistical Analysis with Missing Data, Third Edition. Wiley; 2019.

[32] Powell JR, Gage CB, Crowe RP, Rush LJ, MacEwan SR, Dixon G, et al. National evaluation of emergency medical services clinician burnout and workforce-reducing factors. JACEP Open. 2025. 10.1016/j.acepjo.2024.100024. 40012660 10.1016/j.acepjo.2024.100024 PMC11853008

[33] Remle Patricia Crowe. An Assessment of Burnout among Nationally-Certified Emergency Medical Services. Ohio State University; 2016.

[34] Maslach C, Schaufeli WB, Leiter MP. Job burnout. Annu Rev Psychol. 2001;52:397–422. 11148311 10.1146/annurev.psych.52.1.397

[35] Dong Y, Peng C-YJ. Principled missing data methods for researchers. Springerplus. 2013;2:222. 23853744 10.1186/2193-1801-2-222 PMC3701793

[36] Maslach C, Jackson SE. The measurement of experienced burnout. J Organ Behav. 1981;2:99–113. 10.1002/job.4030020205.

[37] Purvanova RK, Muros JP. Gender differences in burnout: a meta-analysis. J Vocat Behav. 2010;77:168–85.

[38] Roldán GM, Salazar IC, Garrido L, Ramos JM. Violence at work and its relationship with burnout, depression and anxiety in healthcare professionals of the emergency services. Health (San Francisco). 2013;05:193–9.

[39] Rudkjoebing LA, Bungum AB, Flachs EM, Eller NH, Borritz M, Aust B, et al. Work-related exposure to violence or threats and risk of mental disorders and symptoms: a systematic review and meta-analysis. Scand J Work Environ Health. 2020;46:339–49. 31909816 10.5271/sjweh.3877 PMC8506313

[40] Coskun Cenk S. An analysis of the exposure to violence and burnout levels of ambulance staff. Turk J Emerg Med. 2019;19:21–5. https://linkinghub.elsevier.com/retrieve/pii/S2452247318301559 10.1016/j.tjem.2018.09.002 PMC6370911 30793061

[41] Rocha LJ, Cortes MdaCJW, Dias EC, Fernandes FdeM, Gontijo ED. Esgotamento profissional e satisfação no trabalho em trabalhadores do setor de emergência e terapia intensiva em hospital público. Rev Bras Med Trab. 2019;17:300–12. 32368664 10.5327/Z1679443520190404 PMC7195890

[42] Converso D, Sottimano I, Balducci C. Violence exposure and burnout in healthcare sector: mediating role of work ability. Med Lav. 2021;112:58–67. 33635295 10.23749/mdl.v112i1.9906 PMC8023052

[43] World Health Organization (WHO). Global health and care workers compact. 2023. http://apps.who.int/bookorders.

[44] Somani R, Muntaner C, Hillan E, Velonis AJ, Smith P. A systematic review: effectiveness of interventions to de-escalate workplace violence against nurses in healthcare settings. Saf Health Work. 2021;12:289–95. 34527388 10.1016/j.shaw.2021.04.004 PMC8430427

[45] Elsässer A, Dreher A, Pietrowsky R, Flake F, Loerbroks A. Psychosocial working conditions, perceived patient safety and their association in emergency medical services workers in Germany – a cross-sectional study. BMC Emerg Med. 2024;24:62. 10.1186/s12873-024-00983-2. 38616266 10.1186/s12873-024-00983-2 PMC11017549

[46] Zentralinstitut für Arbeitsmedizin und Maritime Medizin (ZfAM). Prävention von Aggressionen und Gewalt gegenüber Beschäftigten in der Notaufnahme. 2023 [cited 2025 Apr 14]. p. 125. https://www.google.com/url?sa=t&source=web&rct=j&opi=89978449&url=https://www.uke.de/dateien/institute/versorgungsforschung-in-der-dermatologie-und-bei-pflegeberufen-(ivdp)/cvcare/gina/neuer-ordner/broschu%25CC%2588re_pra%25CC%2588vention_notaufnahme_einzelseiten_2023.pdf&ved=2ahUKEwi33av_kdiMAxU86wIHHdqhHxYQFnoECBkQAQ&usg=AOvVaw0JwSaccZPW1y5qrI6R2YXC

[47] Statistisches Bundesamt. Fachserie 15 Reihe 4 - Wirtschaftsrechnungen - Private Haushalte in der Informationsgesellschaft - Nutzung von Informations- und Kommunikationstechnologien. 2015 [cited 2019 May 24]. p. 43. https://www.destatis.de/GPStatistik/servlets/MCRFileNodeServlet/DEHeft_derivate_00018504/2150400157004_korr03032016.pdf

[48] Baillargeon J. Characteristics of the healthy worker effect. Occup Med. 2001;16:359–66. 11319057

[49] Podsakoff PM, MacKenzie SB, Podsakoff NP. Sources of method bias in social science research and recommendations on how to control it. Annu Rev Psychol. 2012;63:539–69. 10.1146/annurev-psych-120710-100452. 21838546 10.1146/annurev-psych-120710-100452

