# Supplementary material for: Workplace violence and burnout among emergency medical service workers and nurses in Germany: a cross-sectional study
Source: Hum Resour Health. 2025 Nov 20;23:66. doi: 10.1186/s12960-025-01026-y (PMC12632002; doi:10.1186/s12960-025-01026-y)
Supplement: Supplementary file 1 — Additional file 1. [file 12960_2025_1026_MOESM1_ESM.docx]

**Additional file 1 - Questionnaire**

S1.1 Translation of the MBI

| # of item in original MBI questionnaire | **MBI items** | **German adapted MBI** |
| --- | --- | --- |
| 1 | I feel emotionally drained from my work. | Durch meine Arbeit fühle ich mich gefühlsmäßig am Ende. |
| 2 | I feel used up at the end of the workday. | Am Ende des Arbeitstages fühle ich mich erledigt. |
| 3 | I feel fatigued when I get up in the morning and have to face another day on the job. | Ich fühle mich schon müde, wenn ich morgens aufstehe und wieder ein Arbeitstag vor mir liegt. |
| 4 | I can easily understand how my recipients feel about things. | Es gelingt mir gut, mich in meine Patienten hineinzuversetzen. |
| 5 | I feel I treat some recipients as if they were impersonal ‘objects’. | Ich glaube, ich behandle Patienten zum Teil ziemlich unpersönlich. |
| 6 | Working with people all day is really a strain for me. | Den ganzen Tag mit Leuten zu arbeiten, stellt eine Belastung für mich da. |
| 7 | I deal very effectively with the problems of my recipients. | Mit den Problemen meiner Patienten kann ich gut umgehen. |
| 8 | I feel burned out from my work. | Durch meine Arbeit fühle ich mich ausgelaugt. |
| 9 | I feel I’m positively influencing other people’s lives through my work. | Ich glaube, dass ich das Leben anderer Leute durch meine Arbeit positiv beeinflussen kann. |
| 10 | I’ve become more callous towards people since I took this job. | Seit ich diese Arbeit mache, bin ich gleichgültiger gegenüber Menschen geworden. |
| 11 | I worry that this job is hardening me emotionally. | Ich fürchte, dass diese Arbeit mich emotional verhärtet. |
| 12 | I feel very energetic. | Ich fühle mich voller Tatkraft. |
| 13 | I feel frustrated by my job. | Meine Arbeit frustriert mich. |
| 14 | I feel I’m working too hard on my job. | Ich glaube, ich arbeite zu hart. |
| 15 | I don’t really care what happens to some recipients. | Bei manchen Patienten interessiert es mich eigentlich nicht wirklich, was mit ihnen wird. |
| 16 | Working with people directly puts too much stress on me. | Mit Menschen direkt zusammen zuarbeiten belastet mich zu sehr. |
| 17 | I can easily create a relaxed atmosphere with my recipients. | Es fällt mir leicht, eine entspannte Atmosphäre mit meinen Patienten herzustellen. |
| 18 | I have accomplished many worthwhile things in this job. | Ich habe viele wertvolle Dinge in meiner derzeitigen Arbeit erreicht. |
| 19 | I feel exhilarated after working closely with my recipients. | Ich fühle mich angeregt, wenn ich intensiv mit meinen Patienten gearbeitet habe. |
| 20 | I feel like I’m at the end of my rope. | Ich fühle mich am Ende. |
| 21 | In my work, I deal with emotional problems very calmly. | Ich gehe bei meiner Arbeit mit emotionalen Problemen sehr ruhig und gelassen um. |
| 22 | I feel recipients blame me for some of their problems. | Ich habe das Gefühl, Patienten geben mir die Schuld für einige ihrer Probleme. |
| **Supplement**  Items are grouped by dimensions:   - Emotional exhaustion (9 items; questions 1 – 3, 6, 8, 13, 14, 16, 20), - Depersonalization (5 items; questions 5, 10, 11, 15, 22), - Personal accomplishment (8 items; questions 4, 7, 9, 12, 17 – 19, 21). | | |

S1.2 Items of the RN4Cast and EMS studies

| **Items** | **German adapted version** | **Scale (answer options)** |
| --- | --- | --- |
| EMS: How satisfied are you with your current job?  RN4Cast: How satisfied are you with your current job in this hospital? | EMS/RN4Cast: Wie zufrieden sind Sie alles in allem betrachtet mit Ihrem jetzigen Arbeitsplatz? | 4-point Likert (very dissatisfied, a little dissatisfied, moderately satisfied, very satisfied) |
| EMS: Would you recommend your ward to a colleague as a good place to work?  RN4Cast: Would you recommend your hospital to a nurse colleague as a good place to work? | EMS: Würden Sie Ihren Rettungsdienstbereich einem Kollegen als eine gute Arbeitsstelle weiterempfehlen?  RN4Cast: Würden Sie ihr Krankenhaus einem pflegerischen/r Kollegen/in als eine gute Arbeitsstelle weiterempfehlen? | 4-point Likert (no, probably no, probably yes, yes) |
| EMS: In general, how would you describe the quality of EMS care delivered to patients on your unit/ward?  RN4Cast: In general, how would you describe the quality of nursing care delivered to patients on your unit/ward? | EMS: Wie würden Sie insgesamt die Qualität der rettungsdienstlichen Maßnahmen den Patienten betreffend einstufen?  RN4Cast: Wie würden Sie insgesamt gesehen die Qualität der Pflege, die die Patienten auf Ihrer Station erhalten, einstufen? | 4-point Likert (poor, fair, good, excellent, do not wish to answer) |
| How often would you say each of the following incidents occurs involving you or your patients?   - Complaints from patients or their families - Verbal abuse toward nurses/EMS workers by patients and/or families - Verbal abuse toward nurses/EMS workers by staff) - Physical abuse toward nurses/EMS workers by patients and/or families - Physical abuse toward nurses/EMS workers by staff | Wie oft würden Sie sagen sind die folgenden Ereignisse bei Ihnen eingetreten?   - Beschwerden von Patienten oder Angehörigen - Beschimpfungen der Pflegekräfte/Rettungsdienstkräfte durch Patienten und/oder deren Familienangehörige - Beschimpfungen der Pflegekräfte/Rettungsdienstkräfte durch Mitarbeiter/Kollegen - Handgreiflichkeiten gegenüber Pflegekräften/Rettungsdienstkräften durch Patienten und/oder deren Familienangehörige - Handgreiflichkeiten gegenüber Pflegekräften/Rettungsdienstkräften durch Mitarbeiter/Kollegen | 7-point Likert (Never, a few times, a year or less, once a month or less, a few times a month, once a week, a few times a week, every day, [8-point Likert for EMS: do not wish to answer; EMS]) |

S1.3 Items and scale levels of individual characteristics

| **Item** | **German version** | **Scale and answer options** |
| --- | --- | --- |
| Your sex? | Ihr Alter? | Multiple choice/one answer (male, female) |
| How old are you? | Wie alt sind Sie? | EMS: Multiple choice/one answer; ≤ 19, 20 – 24, 25 – 29, 30 – 34, 35-39, 40 – 44, 45 – 49, 50 – 54, ≥ 55  RN4Cast: Open question/open for input [in years] |
| EMS: What is your highest level of education in EMS?  RN4Cast: What nursing training have you completed? | EMS: Was ist Ihre höchste Ausbildung im Rettungsdienst?  RN4Cast: Welche Pflegeausbildung haben Sie absolviert? | EMS: Paramedic ‘Notfallsanitäter’, paramedic ‘Rettungsassistent’, EMT-I ‘Rettungssanitäter’, EMT-B ‘Rettungshelfer’, medic, open for input  RN4Cast: examinierte Krankenschwester/Pfleger bzw. Gesundheits- und Krankenpfleger, Bachelor of Science in Nursing |
| EMS: How many years have you worked in EMS?  RN4Cast: How many years have you worked as a registered nurse in your career? | EMS: Wie viele Jahre arbeiten Sie bereits im Rettungsdienst?  RN4Cast: Wie viele Jahre haben Sie In Ihrer beruflichen Laufbahn als examinierte/r Pfleger/in gearbeitet? | Open question*/*open for input [in years^d |
| EMS: How is your employment status in emergency medical services?  RN4Cast: Are you working in this hospital full-time? | EMS: Wie sind Sie im Rettungsdienst beschäftigt?  RN4Cast: Sind Sie in diesem Krankenhaus in Vollzeit beschäftigt? | EMS: Full-time, part-time (temporary incl.), voluntary work*  RN4Cast: Multiple choice/one answer (yes, no) |
| * The answer options of the EMS were adapted to the answers of the RN4Cast study. Full-time data was used as a reference and part-time and voluntary work were combined into one category. | | |
