# Supplementary material for: Workplace violence and burnout among emergency medical service workers and nurses in Germany: a cross-sectional study
Source: Hum Resour Health. 2025 Nov 20;23:66. doi: 10.1186/s12960-025-01026-y (PMC12632002; doi:10.1186/s12960-025-01026-y)
Supplement: Supplementary file 2 — Additional file 2. [file 12960_2025_1026_MOESM2_ESM.docx]

# Additional file 2: S2 – Qualifications for EMS workers in Germany

| **Level of EMS education**  **(German term)** | **Content of apprenticeship** |
| --- | --- |
| **Paramedic - 3-year education**  (Notfallsanitäter) | The occupational profile ‘Notfallsanitäter’ was introduced in 2014. Three years full-time apprenticeship. It amends the apprenticeship of the ‘Rettungsassistent’ and includes additional course contents and practical training. |
| **Paramedic - 2-year education**  (Rettungsassistent) | It contains 1.200 hours of a theoretical and hands-on training course in a one-year full-time program and a practical training in an ambulance station for another year full-time (1.600 hours). |
| **EMT-I**  (Rettungssanitäter) | The apprenticeship consists of 160 hours theoretical and 160 hours clinical education plus 160 hours of practice in an ambulance station. Final exam includes a training course and an examination of 40 hours. ¶ |
| **EMT-B**  (most comparable with Rettungshelfer) | The training is based on 160 hours theoretical and practical training and ends plus an examination. An internship for practical training of 80 hours in an ambulance station is mandatory and to be completed in one year. An internship of 80 hours in a hospital is recommended. |
| Based on Baier N, Roth K, Felgner S, Henschke C. Burnout and safety outcomes - a cross-sectional nationwide survey of EMS-workers in Germany. BMC Emerg Med. 2018 Aug 20;18(1):24.  Caption: EMT emergency medical technician, I intermediate, B basic | |
